# Evolution of DFT studies in view of a scientometric perspective

**DOI:** 10.1186/s13321-016-0166-y

**Published:** 2016-10-05

**Authors:** Robin Haunschild, Andreas Barth, Werner Marx

**Affiliations:** 1 Max Planck Institute for Solid State Research, Heisenbergstraße 1, 70569 Stuttgart, Germany; 2 FIZ Karlsruhe - Leibniz Institute for Information Infrastructure, Hermann-von-Helmholtz-Platz 1, 76344 Eggenstein-Leopoldshafen, Germany

**Keywords:** DFT, Chemical bibliometrics, Compound bibliometrics, Publication analysis, RPYS

## Abstract

**Background:**

This bibliometric study aims to analyze the publications in which density functional theory (DFT) plays a major role. The bibliometric analysis is performed on the full publication volume of 114,138 publications as well as sub-sets defined in terms of six different types of compounds and nine different research topics. Also, a compound analysis is presented that shows how many compounds with specific elements are known to be calculated with DFT. This analysis is done for each element from hydrogen to nobelium.

**Results:**

We find that hydrogen, carbon, nitrogen, and oxygen occur most often in compounds calculated with DFT in terms of absolute numbers, but a relative perspective shows that DFT calculations were performed rather often in comparison with experiments for rare gas elements, many actinides, some transition metals, and polonium.

**Conclusions:**

The annual publication volume of DFT literature continues to grow steadily. The number of publications doubles approximately every 5–6 years while a doubling of publication volume every 11 years is observed for the CAplus database (14 years if patents are excluded). Calculations of the structure and energy of compounds dominate the DFT literature.

## Background

Many methods have been developed to solve the Schrödinger [1, 2] and Dirac [3–5] equations approximately. Density functional theory (DFT) has emerged as the most popular one in the past decades. The very first density functional approximation (DFA) was proposed by Thomas [6] and Fermi [7] without mentioning the term. Slater’s [8] simplification of the Hartree–Fock [9, 10] method, the theorems by Hohenberg and Kohn [11], and the orbital-based Kohn–Sham [12] equations mark the beginning of practical DFT calculations. Kohn–Sham-based DFT calculates the energy of a non-interacting reference system and approximates the difference to the real system using the exchange and correlation functionals. The first exchange and correlation functionals depend only on the electron density itself [13]. More accurate calculations became possible with the development of exchange and correlation functionals that also included the gradient (GGA functionals) [14–17] and second derivatives (meta-GGA functionals) [18–20] of the electron density. These provide increased accuracy at negligible additional computational expense. The development of hybrid functionals [21–26] marked the point where the accuracy and popularity of DFT increased dramatically. At first, some amount of Hartree–Fock exchange was admixed with the exchange functional. Higher accuracy for atoms and molecules as well as applicability of hybrid functionals to solids and surfaces was achieved by range-separation [27–34]. The development of local hybrids [35–41] increased the accuracy of DFT calculations further. The concepts of local hybrids and range-separation were then combined [42–44] yielding even more accurate results. As hybrid functionals became popular, correlation from wave function methods (MP2 [45, 46], RPA [47–50], coupled-cluster [51–53]) was also admixed with the correlation functional [54, 55].

Individual researchers in the field of DFT do have a qualitative overview about publications related to DFT and compounds computed with DFT, but a quantitative overview can only be obtained using bibliometric methods. Although there is considerable interest in the evolution of the annual publication volume in the field of DFT [56, 57], no detailed bibliometric study was published about DFT publications so far. We intend to fill the gap with this study.

Bibliometrics or the broader term, scientometrics—both terms are often used synonymously—can be characterized as the discipline that treats science quantitatively [58, 59]. Publication and citation numbers are the most important items that have become the basis of bibliometric indicators for research evaluation purposes. In many disciplines, particularly in chemistry, physics, and materials science, chemical compounds (substances) play a major role. In a previous paper [60], we have extended the bibliometric method and defined compound-based (chemical) bibliometrics as a new research field. The method can be applied to analyze large numbers of publications and compounds in combination with the corresponding chemical concepts: We can establish the time evolution of the publications dealing with concepts or methods and reveal the related compounds or compound classes. Furthermore, the mapping of method related compounds by establishing element-based landscapes has some potential to illustrate the compound basis of research topics.

Reference Publication Year Spectroscopy (RPYS) is a bibliometric method which can be used to locate seminal papers which are cited most frequently in a certain publication set [61]. The method is based on the analysis of cited references (i.e. the number of times a specific reference is included the reference lists) in published papers of certain scientific fields. Researchers in the field can answer the question about seminal papers only subjectively. RYPS can answer this question in an objective way by asking all researchers in the field (via the cited references in their publications) with subsequent quantitative analysis. Therefore, RPYS results often provide a different perspective or complement the individual expert’s perspective on the field.

## Methods

Our analysis is based on the search and retrieval functions of the databases offered by Chemical Abstracts Service (CAS), a division of the American Chemical Society (ACS). The CAS literature database (Chemical Abstracts Plus, CAplus^SM^) covers scientific publications and patents since around 1900 (including the references cited therein since the publication year 1996). The CAS compound database (Registry^SM^) contains all chemical species mentioned within the publications in chemistry and related fields, identified and registered by the CAS Registry system. All compound records are associated with a unique CAS Registry number. These items (publications and compounds) are called documents or records. We used both databases via the new platform of the Scientific and Technical Information Network (STN^®^) International. Both databases are connected to each other via Registry numbers^®^ (RNs). The content of both databases is also accessible with SciFinder^®^. However, the STN platform provides more detailed search and analysis possibilities.

The CAplus publication records contain index terms (ITs, keywords carefully selected and assigned by the database producer CAS). We searched for the terms “DFT”, “density functional theory”, “d functional theory”, and “TDDFT” in the IT fields of the CAplus database. Occurrences of “TD-DFT” and “time-dependent density functional theory” are also found by our aforementioned search terms. The search term “d functional theory” is not used by scientists using DFT but it is used by CAS indexers. In total, we found 114,138 documents published before the end of the year 2014 (at the date of searching the year 2015 was not completely covered by the database). Throughout this paper, we will refer to this set of 114,138 documents as “all DFT publications”. Although indexing takes some time, we can expect that the publication years until 2014 are nearly complete. 102,880 documents (90.1 %) have at least one connection to a Registry compound record. Throughout this paper, we will refer to this set of 102,880 documents as “substance-related DFT publications”. The compounds with at least one connection from a Registry to a CAplus record will be referred to as “DFT-related compounds”. The remaining 9.9 % of the documents are either concerned with methodological developments or the calculated substances are not a major concern of the document.

We used the relationship between CAplus and Registry mainly to elucidate how often which elements are present in the corresponding compounds connected to DFT calculations. An example of the CAplus IT fields is shown in Table 1 using the document in Ref. [62].

**Table 1:** IT fields and index terms as an example for CAS indexing of the document in Ref. [62] where DFT was applied to a set of molecules

IT field	Index terms
IT1	1202652-94-5, 1202652-95-6, 1202652-96-7, 1202652-97-8, 1202652-98-9, 1202652-99-0, 1202653-00-6, 1202653-01-7, 1202653-02-8, 1202653-03-9, 1202653-04-0, 1202653-05-1, 1202653-06-2, 1202653-07-3, 1202653-08-4, 1202653-09-5, 1202653-10-8, 1202653-11-9, 1202653-12-0, 1202653-13-1, 1202653-15-3, 1202653-16-4, 1202653-17-5, 1202653-18-6, 1202653-19-7, 1202653-20-0, 1202653-21-1, 1202653-22-2, 1202653-23-3, 1202653-24-4, 1202653-25-5, 1202653-26-6, 1202653-27-7, 1202653-28-8, 1202653-29-9, 1202653-30-2, 1202653-31-3, 1202653-32-4, 1202653-33-5, 1202653-34-6, 1202653-35-7, 1202653-36-8, 1202653-37-9, 1202653-38-0, 1202653-39-1, 1202653-40-4, 1202653-41-5, 1202653-42-6, 1202653-43-7, 1202653-44-8, 1202653-45-9, 1202653-46-0, 1202653-47-1, 1202653-48-2, 1202653-49-3, 1202653-50-6, 1202653-51-7, 1202653-52-8, 1202653-53-9, 1202653-54-0, 1202653-55-1 Properties (PRP) Dewar–Chatt–Duncanson model reversed and bonding anal. of Ni, Pd, and Pt complexes [(PMe_3_)_2_M-EX_3_] with Group IIIA element E halide ligands EX_3_ from DFT-BP86 calcns
IT2	Conformation Dissociation energy Bond, coordinate Dewar–Chatt–Duncanson model reversed and bonding anal. of Ni, Pd, and Pt complexes [(PMe_3_)_2_M-EX_3_] with Group IIIA element E halide ligands EX_3_ from DFT-BP86 calcns
IT3	Electron density Partial charges; Dewar–Chatt–Duncanson model reversed and bonding anal. of Ni, Pd, and Pt complexes [(PMe_3_)_2_M-EX_3_] with Group IIIA element E halide ligands EX_3_ from DFT-BP86 calcns
IT4	Molecular structure Dewar–Chatt–Duncanson model reversed and bonding anal. of Ni, Pd, and Pt complexes [(PMe_3_)_2_M-EX_3_] with Group IIIA element E halide ligands EX_3_ from DFT-BP86 calcns
IT5	Potential energy Decompn. anal.; Dewar–Chatt–Duncanson model reversed and bonding anal. of Ni, Pd, and Pt complexes [(PMe_3_)_2_M-EX_3_] with Group IIIA element E halide ligands EX_3_ from DFT-BP86 calcns
IT6	Transition metal complexes Properties (PRP) Dewar–Chatt–Duncanson model reversed and bonding anal. of Ni, Pd, and Pt complexes [(PMe_3_)_2_M-EX_3_] with Group IIIA element E halide ligands EX_3_ from DFT-BP86 calcns

For example, the first index term (IT1) shown in Table 1 contains the relevant compounds in the form of their RNs together with the controlled term “Properties (PRP)” in combination with the corresponding abbreviated author vocabulary (“Dewar–Chatt–Duncanson model reversed and bonding anal. of Ni, Pd, and Pt complexes [(PMe_3_)_2_M-EX_3_] with Group IIIA element E halide ligands EX_3_ from DFT-BP86 calcns.”). This indicates that properties were calculated for the substances that correspond to the itemized RNs and are described by the abbreviated author vocabulary. The other IT fields contain additional combinations of controlled terms with abbreviated author vocabulary.

We use controlled terms supplied by the indexer (e.g., “Molecular structure”, “Conformation”, “Bond”) to define sub-fields or topics within the corpus of DFT literature. The topics together with carefully selected index terms are presented in Table 2.

**Table 2:** Topics within the DFT literature as defined by carefully selected index terms

Topic	Index terms
Structure	Molecular structure; Bond length; Bond angle; Crystal structure; Surface; Aromaticity; Antiaromaticity; QSPR (quantitative structure–property relationship); Ring current (molecular); Transition state structure; Lattice parameters; Conformation; Structure–activity relationship; Protein conformation; Peptides; Molecular topology; Solvent polarity effect; Steric effects; Substituent effects; Tautomers; Dissociation; Crystal orientation
Energy	Excited state; Ground state; Excited vibrational state; Molecular rotation; Vibrational energy; Vibrational frequency; Molecular vibration; Rotational transition; Adsorption; Binding energy; Energy level; Total energy; Zero point energy
Spectroscopy	IR spectra; Fluorescence; Absorption; Chromophore; Photoelectron spectra; NMR; Nuclear magnetic resonance; Nuclear shielding; NICS; Nucleus-independent chemical shifts; Spin-rotation coupling; Spin–spin coupling; Hyperfine coupling; Hyperfine splitting; Microwave spectra
Electronic properties	Electronic properties; Charge Transfer; Electric field gradient; Quadrupole coupling; Quadrupole moment; Dipole moment; Hyper polarizability; Hyperpolarizability; Polarizability; Optical hyperpolarizability; Third-order nonlinear optical properties; Electronic structure; Hardness (electronic structure); Softness (electronic structure)
Thermodynamics	Heat capacity; Free energy function; Adsorption; Enthalpy; Entropy; Free energy
Chemical bond	Bond; Noncovalent bond; Covalent bond; Ionic bond; Electron affinity; Bond order; Hydrogen bond
Reactions	Reactions; Reaction mechanism; Reduction; Reduction catalysts; Addition reaction; Rearrangement; Isomerization; Reaction mechanism; Conformational transition; Hydrothermal reaction; Thermal decomposition; Substitution reaction; Potential energy surface; Tautomerization; Activation energy; Proton transfer; Potential barrier
Relativity	Relativity; ZORA; Zeroth-order regular approximation; Spin–orbit coupling; Two-component; Four-component; Relativistic
Magnetism	Antiferromagnetic exchange; Antiferromagnetic materials; Antiferromagnetic; Anti-ferromagnetic; Ferromagnetic; Magnetic susceptibility

We also analyze the DFT publications with respect to seminal papers on which the DFT publications are based. Such seminal papers can be located using a bibliometric method called “Reference Publication Year Spectroscopy” (RPYS) [61] in combination with a recently developed tool named CRExplorer (http://www.crexplorer.net) [63]. The analysis of the publication years of the references cited by all the papers in a specific research field shows that (earlier) publication years are not equally represented. Some years occur particularly frequently among the references. The years appear as pronounced peaks in the distribution of the reference publication years (i.e. the RPYS spectrum). The peaks are frequently based on single early publications, which are highly cited compared to other early publications. The highly cited papers are usually of specific significance to the research field in question (here: DFT).

In a first step, the publication set is imported into the CRExplorer and all cited references are extracted. In a second step, equivalent references are clustered and merged. References below a threshold (here: 100 cited references) are removed to reduce the background noise and to sharpen the resulting spectrum. In the third and final step, the reference publication years are analyzed for frequently cited publications. We analyze the reference publication years (RPYs) between 1950 and 1990. It is very problematic to analyze younger RPYs than 1990, and 1950 is a reasonable choice as the oldest RPY for the topic DFT. Furthermore, older RPYs require a slightly different methodology, i.e., lower threshold of the number of cited references.

## Results

### Overall growth and growth in terms of topics

**Fig. 1:**
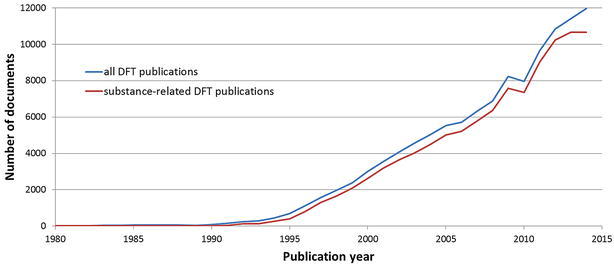
Overall annual volume of DFT publications since 1980

The overall annual publication volume since 1980 that is concerned with DFT is shown in Fig. 1. Note that 13 DFT relevant publications (11 substance-related DFT publications) were published prior to 1980.

According to Fig. 1, the annual publication volume shows a strong increase since 1995. The curve of all DFT publications (blue line) is nearly parallel to the curve of DFT publications with a connection to a RN (substance-related DFT publications, red line) until 2012. Probably, the indexing of the recent years still needs some time to be completed so the years 2013 and 2014 should be looked at with caution. The annual volume of DFT publications shows a doubling within 5–6 years, which is much faster than the overall growth of the CAplus literature. The total volume of publications covered by CAplus between 1968 (first publication with DFT-related index term) and 2013 doubled approximately only every 11 years (14 years when patents are excluded). The growth rate of DFT literature is, for example, comparable to the growth rate of literature for a hot topic like climate change [64].

**Fig. 2:**
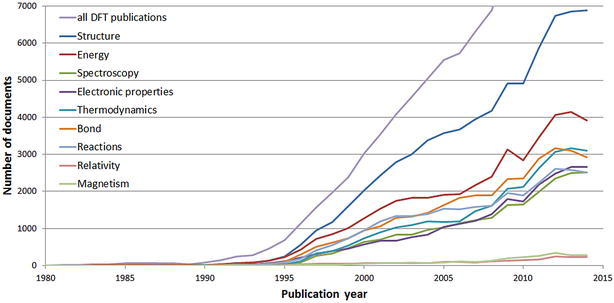
Growth of DFT publications in terms of research topics since 1980. These research topics comprise 98,357 documents. The curve for all DFT publications is included for comparison

Figure 2 shows the growth of DFT publications in terms of research topics since 1980. Note that there is of course some overlap between the research topics.

Nearly all the topic curves in Fig. 2 show a decline or slowed growth rate in the years 2013 and 2014, just as the red curve showed in Fig. 1. This effect is probably also attributable to the delayed indexing for the recent years. The topics “Structure” and “Energy” start to increase before the other topics. As Fig. 2 indicates, index terms related to the topic “Energy” are only included in the record if the determination of the energy plays an essential role in the publication. The index terms related to the topic “Energy” are not included in the record if the energy calculation is only necessary to obtain properties of substances. In order to calculate the structure of a substance, obviously, one has to calculate the energy first. In such instances, the index terms related to the topic “Energy” are not added to the list of index terms. “Relativity” and “Magnetism” increase at a much slower rate than the other topics. The nine topics comprise 86.5 % of all DFT publications and 95.6 % of the substance-related DFT publications.

### Substance-related analysis of DFT literature

**Fig. 3:**
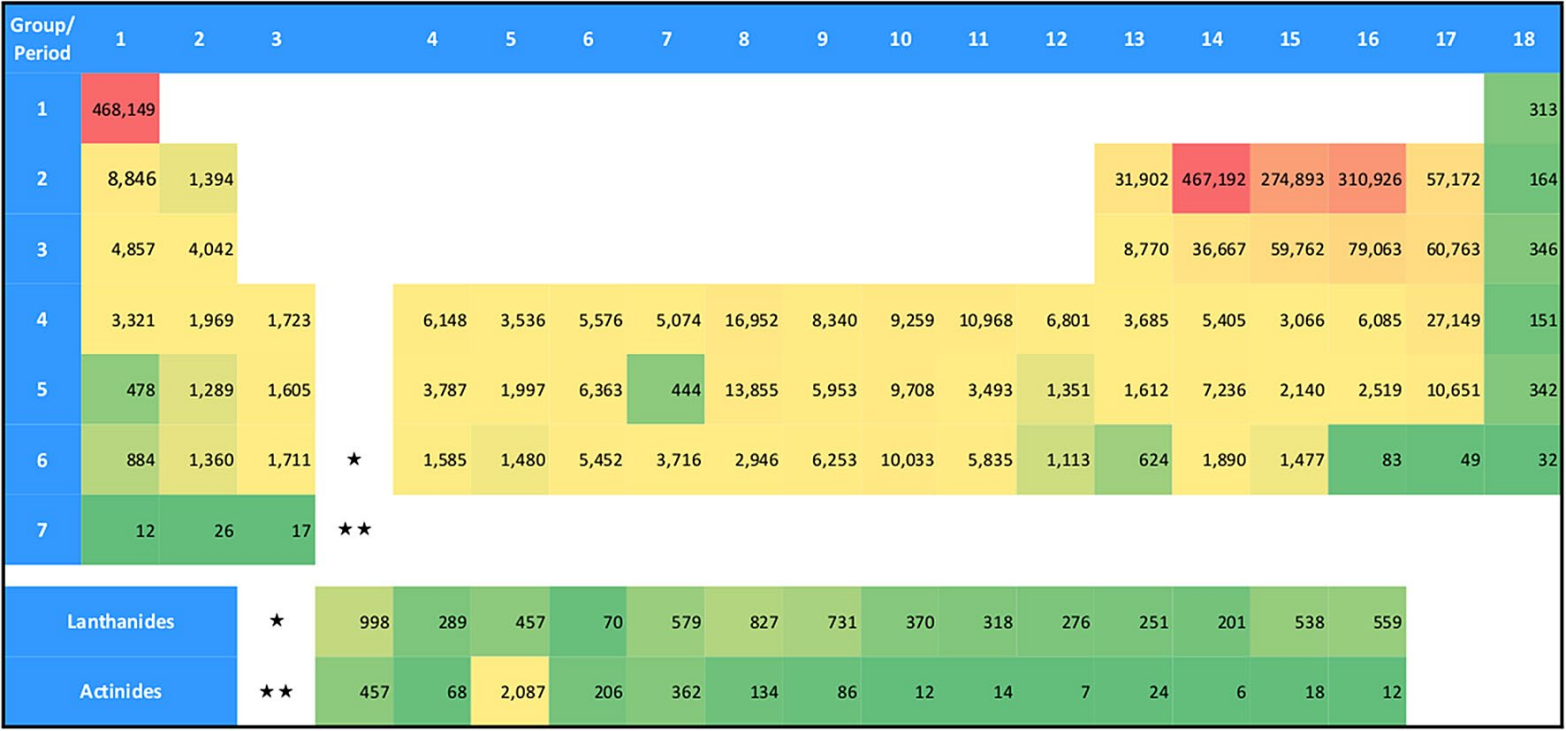
Periodic table shown as a heat map containing the number of compounds mentioned within our publication set of DFT literature (DFT-related publications). The cells are color-coded: from *red* (very high occurrence) over *orange* and *yellow* to *green* (very low occurrence)

For the substance-related analysis, we extracted all Registry numbers from the publication set of all DFT papers (n = 114,138) and transferred them to the compound database Registry. The records of the compound database include various compound specific information, in particular the chemical names, molecular formulas, and structure diagrams. The search for the number of compounds indexed in DFT literature and containing specific elements was based on the molecular formula field. We determined how many compounds containing a specific element have been indexed within the DFT publication set. Figure 3 shows a periodic table where instead of the element symbols the absolute number of compounds within the DFT literature is given. It is important to note that numbers in the table may overlap, e.g. between C (467,192) and O (274,893).

By far the most frequently occurring elements in compound-specific publications dealing with DFT calculations are hydrogen and carbon. Oxygen and nitrogen also occur very often in substance-related DFT calculations. The lanthanides and actinides occur about as often in compound-specific DFT calculations as the rare gas elements, with one exception: uranium occurs significantly more often than the other actinides.

**Fig. 4:**
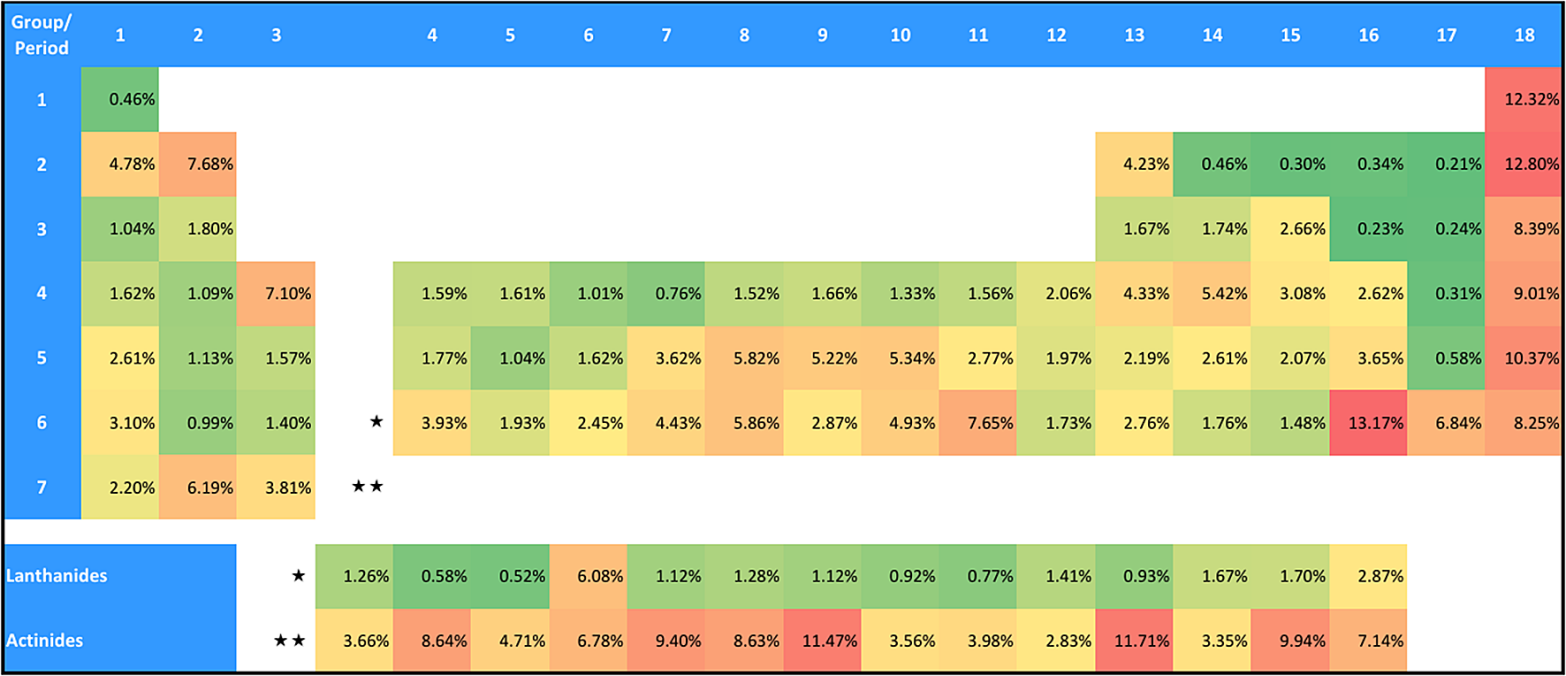
Percentage of compounds with DFT-related publications relative to all compounds registered at CAS. The cells are color-coded: from *red* (very high occurrence) over *orange* and *yellow* to *green* (very low occurrence)

Figure 4 shows the percentage of compounds that have DFT-related publications registered relative to all registrations for each specific element. Although the absolute numbers in Fig. 3 are rather low, the percentages of DFT-related compounds are quite high for the rare gases, many actinides, and polonium. Also, some transition metals (e.g., gold, platinum, palladium, rhodium, ruthenium, and osmium) show rather high relative occurrences. Figure 3 shows very high absolute numbers for hydrogen, carbon, nitrogen, and oxygen whereas Fig. 4 shows that their relative share of DFT-related compounds is rather low.

**Fig. 5:**
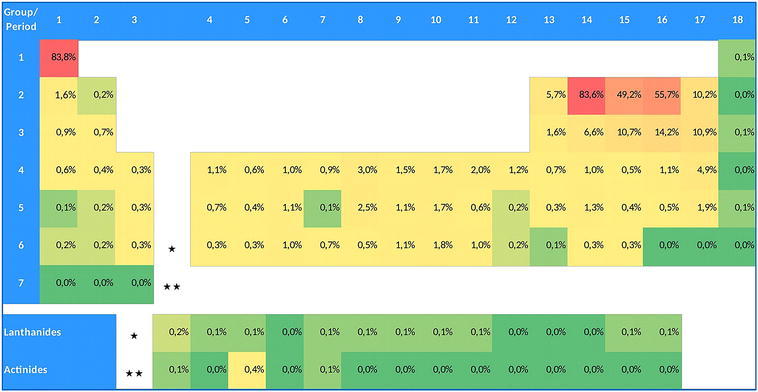
Percentage of compounds with DFT-related publications relative to all DFT-related compounds (n = 558.619). The cells are color-coded: from *red* (very high occurrence) over *orange* and *yellow* to *green* (very low occurrence)

In total, 558.619 DFT-related compounds were found. Figure 5 shows the share of each element relative to the total of 558.619 DFT-related compounds. The color-coding is essentially the same as in Fig. 3.

**Fig. 6:**
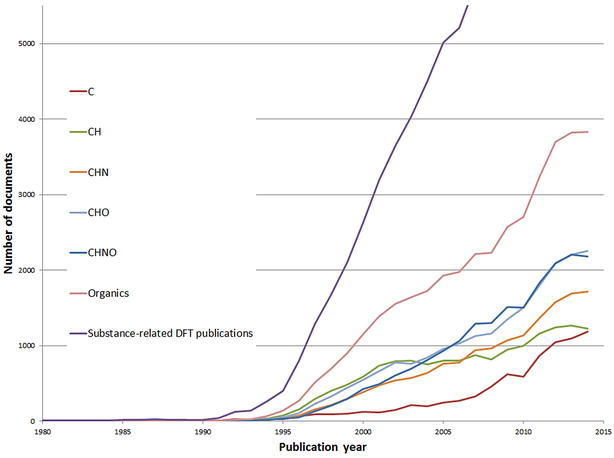
Annual publication volume in terms of DFT studies that investigate compounds containing certain elements centered on carbon-containing compounds

Figure 6 shows the annual publication volume of DFT studies that investigate compounds containing certain elements centered on carbon-containing compounds. Only the elements shown are allowed to occur in the sum formula (e.g. in the case of C no elements other than carbon are allowed in the sum formula, CH indicates pure hydrocarbons, etc.). Organics is the super-set of CH, CHN, CHO, and CHNO. Of course, there are more organic compounds, but this analysis concentrates on pure organic compounds and excludes compounds with less common hetero-atoms. For comparison, also the total curve of all substance-related DFT publications is included in the Figure. The first substance indexed with these element restrictions was a carbon modification published in 1982.

Most of the compounds contributing to the C curve are fullerenes. Additionally, different oxidation states and isotopes of the carbon atom are registered as different compounds. The curves of CHN, CHO, and CHNO are very similar. Probably, the reason is that O and NH are isoelectronic. Therefore, most CHO compounds can also be calculated when oxygen is substituted by an NH group. The curve “Organics” (according to our definition) covers 37.2 % (n = 38,277 papers) of the substance-related DFT literature. Again, the decline or slowed growth rate in the years 2013 and 2014 is probably caused by the delayed indexing for the recent publication years.

**Fig. 7:**
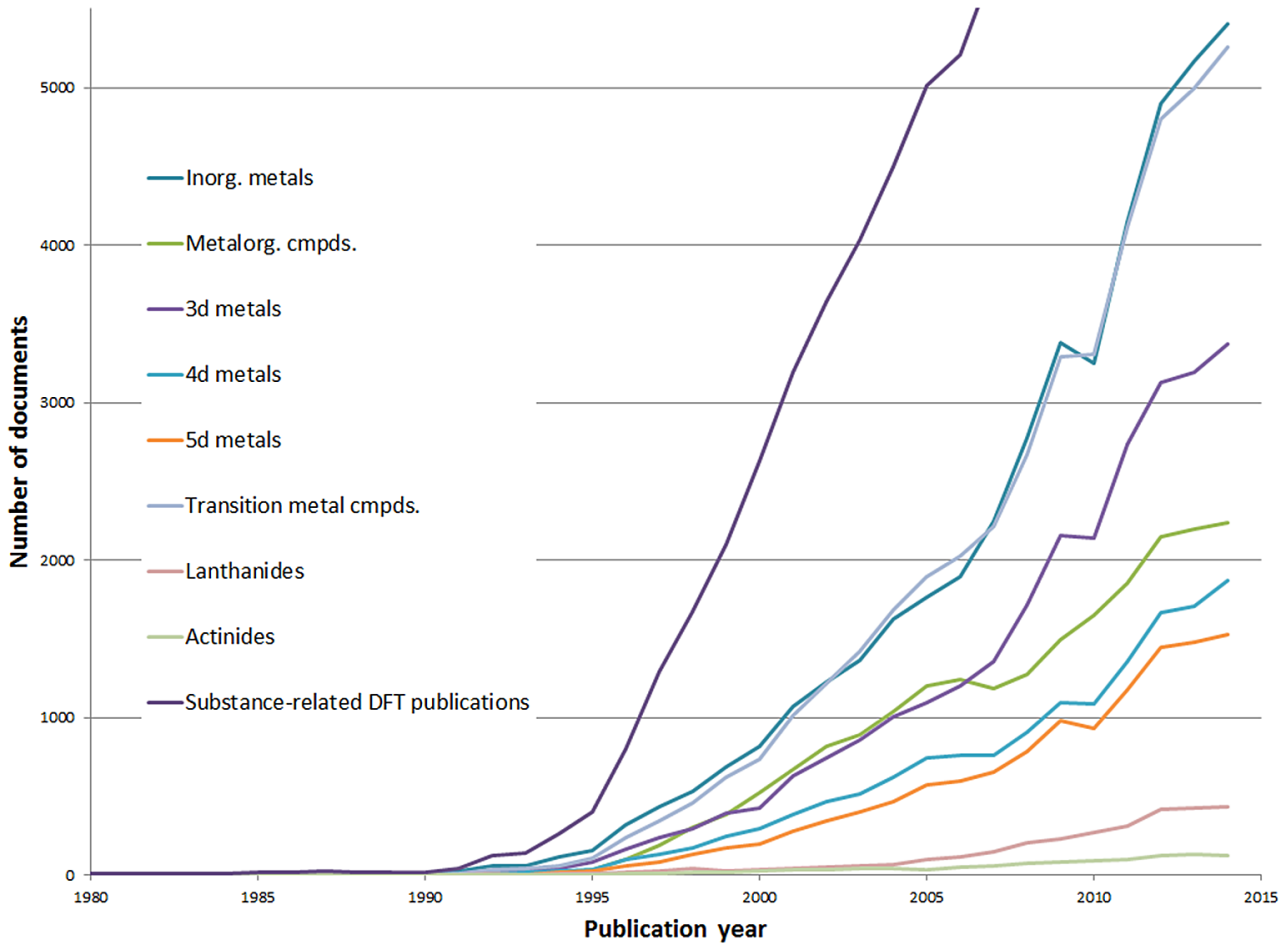
Annual publication volume in terms of DFT studies that investigate specific compound groups

Figure 7 shows the annual publication volume of DFT studies that investigate specific compound groups: inorganic metals, organometallic compounds, transition metal compounds, lanthanides, and actinides. Here, organometallic compounds are defined as a compound with at least one metal, carbon, and hydrogen atom. There is no restriction on additional elements. For comparison, also the total curve of all substance-related DFT publications is included in the Figure. The compound paper curves in Figs. 5 and 6 cover 81.6 % of the total set of all substance-related DFT publications.

The largest compounds calculated with DFT in terms of number of atoms are: C_6000_ [65], C_5120_ [66], and C_4860_ [65]. All three compounds are fullerenes with icosahedral symmetry. Unfortunately, the Registry database does not have point groups as additional information for the registered molecules, so one cannot search for the largest asymmetric molecule calculated with DFT. Also, the information about employed basis sets and specific density functionals is often missing in the CAplus database. Therefore, it is not possible using our search strategy to find the computationally most demanding molecule calculated with DFT.

### Analysis of seminal DFT papers

**Fig. 8:**
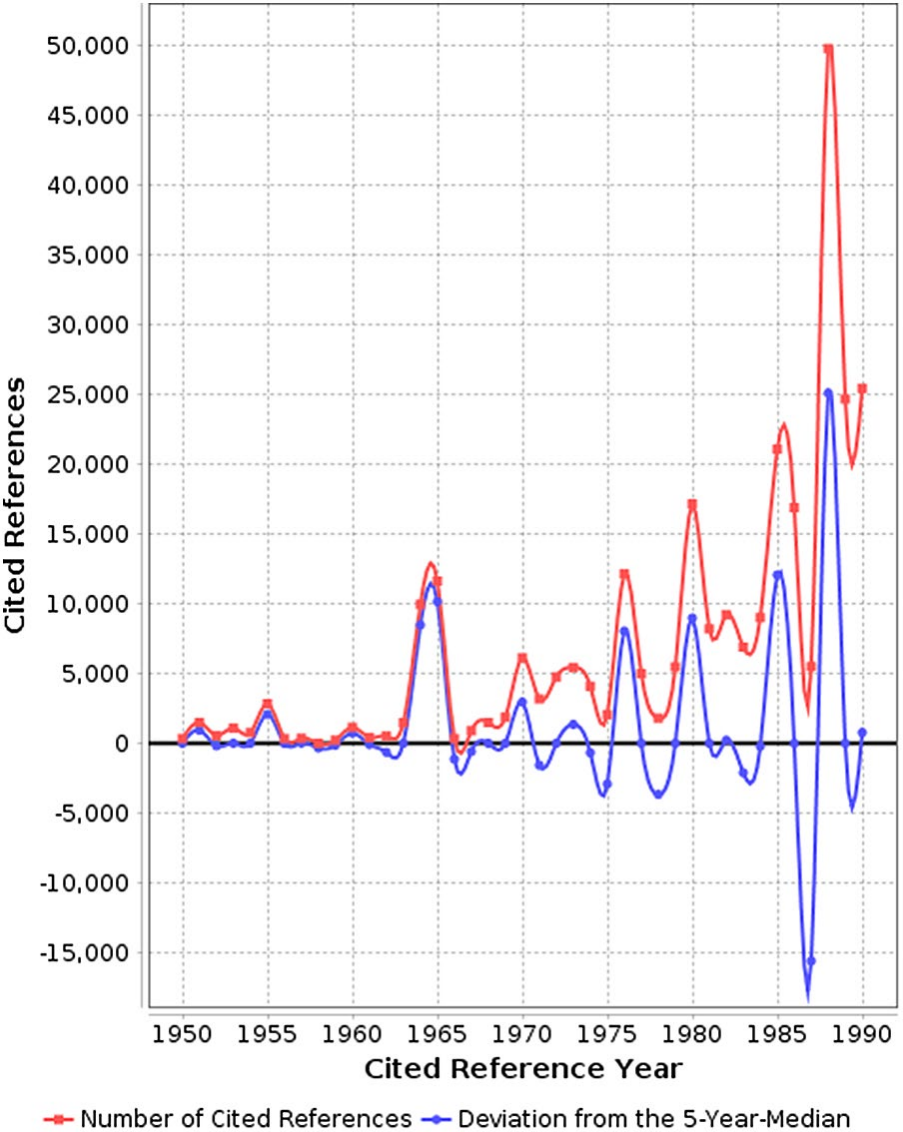
Annual distribution of the references cited in DFT publications across their reference publication years within the time period 1950-1990. Only references with a minimum reference count of 100 are considered here

Figure 8 shows the result of the RPYS performed with the CRExplorer. The figure presents the distribution of the number of cited references across their publication years within the time period 1950–1990. Nine distinct peaks (1951, 1955, 1964/1965, 1970, 1972/1973, 1976, 1980, 1986, and 1988) can be located in the spectrum. The publications which are mainly responsible for these peaks are listed in Table 3. The red line in Fig. 8 visualizes the number of cited references per reference publication year. In order to identify those publication years with significantly more cited references than other years, the (absolute) deviation of the number of cited references in each year from the median of the number of cited references in the two previous, the current, and the two following years (t − 2; t − 1; t; t + 1; t + 2) is also visualized (blue line). This deviation from the 5-year median provides a curve smoother than the one in terms of absolute numbers. We used both curves for the identification of the peaks. Table 3 contains the seminal papers which are mainly responsible for the peaks. This is a highly selective method and many other seminal papers relevant to DFT are not mentioned in Table 3. However, such papers can be identified via the reference table alongside the spectrum in the CRExplorer.

**Table 3:** The most frequently cited references from specific reference publication years cited by DFT publications

N^o^	RPY	Cited reference	NCR
CR1	1951	Slater J, 1951, Phys Rev, V81, P385	737
CR2	1951	Roothaan C, 1951, Rev Mod Phys, V23, P69	381
CR3	1955	Mulliken R, 1955, J Chem Phys, V23, P1833	1700
CR4	1964	Hohenberg P, 1964, Phys Rev B, V136, P864	8213
CR5	1965	Kohn W, 1965, Phys Rev A, V140, P1133	9634
CR6	1970	Boys S, 1970, Mol Phys, V19, P553	3196
CR7	1972	Hehre W, 1972, J Chem Phys, V56, P2257	2659
CR8	1973	Hariharan P, 1973, Theor Chim Acta, V28, P213	3001
CR9	1973	Baerends E, 1973, Chem Phys, V2, P41	1258
CR10	1976	Monkhorst H, 1976, Phys Rev B, V13, P5188	6506
CR11	1980	Vosko S, 1980, Can J Phys, V58, P1200	6046
CR12	1986	Perdew J, 1986, Phys Rev B, V33, P8822	6106
CR13	1988	Lee C, 1988, Phys Rev B, V37, P785	23,953
CR14	1988	Becke A, 1988, Phys Rev A, V38, P3098	14,150

For each cited reference, a sequential number (N^o^), the corresponding reference publication year (RPY), and the number of cited references (NCR) within the publication set are listed

The cited references CR1, CR4, CR5, and CR11–CR14 of Table 3 were mentioned in the Background Section of this study. Four of them (CR11–CR14) propose new density functional approximations or improvements to existing ones. The cited references CR4 and CR5 are the foundational publications for modern DFT by Hohenberg and Kohn (CR4) and Kohn and Sham (CR5). The cited reference CR1 is Slater’s approximation to Hartree–Fock exchange. The seven other cited references in Table 3 are not specific about DFT. They are of a more general interest in theoretical and computational chemistry and physics.

In cited reference CR2 Roothaan proposes to construct molecular orbitals as a linear combination of atomic orbitals (LCAO). This proposal was made for Hartree–Fock theory but is used in virtually every widespread program package for post-Hartree–Fock and DFT calculations. In cited reference CR3 Mulliken proposed an electronic population analysis based on Roothaan’s LCAO method. Using this methodology, it became possible to calculate partial charges and dipole moments.

Boys and Bernardi proposed in cited reference CR6 a new direct difference method for the computation of molecular interaction energies with reduced errors. Hehre, Ditchfield, and Pople presented new basis sets for the LCAO method in reference CR7. The 6-31G basis set, which became very popular, is among those basis sets presented in this cited reference. The relevance of polarization functions was pointed out by Hariharan and Pople in cited reference CR8, and the popular 6-31G* and 6-31G** basis sets were proposed. Baerends, Ellis, and Roos presented in cited reference CR9 a computational Hartree–Fock scheme using Slater’s approximation and Roothaans LCAO ansatz where Slater-type atomic orbitals do not increase the computational demand compared to Gaussian-type orbitals. Cited reference CR10 by Monkhorst and Pack is the only cited reference in Table 3 concerned specifically with the solid state. They propose a method for generating sets of special points in the Brillouin zone. This method provides a more efficient algorithm to integrate periodic functions of the wave vector in solid state calculations.

## Discussion

Most DFT literature is substance-related. Therefore, the publication volumes of the general DFT literature are very similar to the publication volumes of the substance-related DFT literature. In terms of absolute numbers, most compounds calculated by DFT contain hydrogen, carbon, nitrogen, or oxygen. Also, 37.2 % of the substance-related DFT literature is concerned with compounds build from these four elements. 81.6 % of the substance-related DFT literature is covered when broader compound groups (inorganic metals, organometallic compounds, transition metal compounds, lanthanides, and actinides) are considered additionally. However, a relative perspective shows that DFT calculations were performed rather often in comparison with experiments for rare gas elements, many actinides, and polonium as well as some transition metals. Probably, we see rather high activity of DFT research for many actinides and polonium because of industrial interest in combination with interest in their radioactive decay. The interest in platinum, palladium, rhodium, ruthenium, and osmium might be due to their catalytic activity. The highly selective RPYS analysis shows the 14 most influential publications with relevance to DFT published between 1950 and 1990. Seven of these 14 publications were cited in the Background section of this manuscript. The other DFT publications cited in the Background section of this manuscript are newer or older.

We have to mention here the limitations of our study. Our retrieval strategy, based only on index terms, can be seen as a limitation as we obtain fewer publications this way than by a search in title, keywords, and abstract for DFT-related keywords. This strategy is a compromise to gather the publications where DFT plays a major role. Another search strategy would yield too many false positives, i.e. publications where DFT plays only a minor role although DFT-related keywords are mentioned in title, keywords, or abstract. In contrast to previous chemical bibliometric studies, we did not use only hit RNs but all RNs in the records. This is necessary because control tests showed that too few RNs were supplemented with DFT-related terms. However, we can assume that these limitations do not change the picture.

The RPYS analysis has certain additional limitations. There are also seminal DFT papers published before 1950 and after 1990. However, reference publication years younger than 1990 require a different technical treatment because of the exponential increase of the number of publications and cited references. Seminal papers before 1950 comprise the historical roots of DFT and are an interesting subject for another analysis. We chose to be highly selective in the identification of seminal papers in the DFT literature. A less selective procedure would result in many more seminal papers. Such a detailed analysis is possible but is beyond the scope of our current analysis.

Of course, our search and analysis strategy is not limited to the topic DFT. Similar bibliometric analyses can be performed for other topics where a connection between publications and chemical substances is important.

## Conclusions

In conclusion, we have presented an overview of the total and compound-related DFT literature. The total DFT literature was analyzed in terms of research topics while the compound-related DFT literature was analyzed in terms of chemical elements and combinations of elements. DFT-related literature experienced an exponential growth during the 1990s. Since 2000 the growth of DFT-related publications has become linear. Currently, the DFT publication volume doubles every 5–6 years. Finally, we have identified and discussed 14 seminal papers of the DFT literature.

## Abbreviations

TDDFT: time-dependent density functional theory
DFT: density functional theory
CAS: Chemical Abstracts Service
CAplus^SM^: Chemical Abstracts Plus
ACS: American Chemical Society
STN: Scientific and Technical Information Network
RNs: Registry numbers

## References

[1] Schrodinger E (1926). Quantisation as an eigen value problem. Ann Phys.

[2] Schrodinger E (1926). An undulatory theory of the mechanics of atoms and molecules. Phys Rev.

[3] Dirac PAM (1928). The quantum theory of the electron. Proc R Soc Lond Ser A Contain Pap Math Phys Character.

[4] Dirac PAM (1928). The quantum theory of the electron—part II. Proc R Soc Lond Ser A Contain Pap Math Phys Character.

[5] Dirac PAM (1928). On the quantum theory of electrons. Physikalische Zeitschrift.

[6] Thomas LH (1927). The calculation of atomic fields. Proc Camb Philos Soc.

[7] Fermi E (1928). A statistical method for determining some properties of the atoms and its application to the theory of the periodic table of elements. Z Angew Phys.

[8] Slater JC (1951). A simplification of the Hartree–Fock method. Phys Rev.

[9] Hartree DR, Hartree FRS, Hartree W (1935). Self-consistent field, with exchange, for beryllium. Proce R Soc Lond Ser Math Phys Sci.

[10] Fock V (1930). Approximation method for the solution of the quantum mechanical multibody problems. Z Angew Phys.

[11] Hohenberg P, Kohn W (1964). Inhomogeneous electron gas. Phys Rev B.

[12] Kohn W, Sham LJ (1965). Self-consistent equations including exchange and correlation effects. Phys Rev.

[13] Vosko SH, Wilk L, Nusair M (1980). Accurate spin-dependent electron liquid correlation energies for local spin-density calculations—a critical analysis. Can J Phys.

[14] Perdew JP (1986). Density-functional approximation for the correlation-energy of the inhomogeneous electron-gas. Phys Rev B.

[15] Perdew JP, Burke K, Ernzerhof M (1996). Generalized gradient approximation made simple. Phys Rev Lett.

[16] Becke AD (1988). Density-functional exchange-energy approximation with correct asymptotic-behavior. Phys Rev A.

[17] Lee CT, Yang WT, Parr RG (1988). Development of the Colle–Salvetti correlation-energy formula into a functional of the electron-density. Phys Rev B.

[18] Tao JM, Perdew JP, Staroverov VN, Scuseria GE (2003). Climbing the density functional ladder: nonempirical meta-generalized gradient approximation designed for molecules and solids. Phys Rev Lett.

[19] Perdew JP, Kurth S, Zupan A, Blaha P (1999). Accurate density functional with correct formal properties: a step beyond the generalized gradient approximation. Phys Rev Lett.

[20] Zhao Y, Truhlar DG (2006). A new local density functional for main-group thermochemistry, transition metal bonding, thermochemical kinetics, and noncovalent interactions. J Chem Phys.

[21] Becke AD (1993). Density-functional thermochemistry. 3. The role of exact exchange. J Chem Phys.

[22] Adamo C, Barone V (1999). Toward reliable density functional methods without adjustable parameters: the PBE0 model. J Chem Phys.

[23] Zhao Y, Schultz NE, Truhlar DG (2005). Exchange-correlation functional with broad accuracy for metallic and nonmetallic compounds, kinetics, and noncovalent interactions. J Chem Phys.

[24] Zhao Y, Schultz NE, Truhlar DG (2006). Design of density functionals by combining the method of constraint satisfaction with parametrization for thermochemistry, thermochemical kinetics, and noncovalent interactions. J Chem Theory Comput.

[25] Becke AD (1993). A new mixing of Hartree–Fock and local density-functional theories. J Chem Phys.

[26] Perdew JP, Emzerhof M, Burke K (1996). Rationale for mixing exact exchange with density functional approximations. J Chem Phys.

[27] Heyd J, Scuseria GE, Ernzerhof M (2003). Hybrid functionals based on a screened Coulomb potential. J Chem Phys.

[28] Heyd J, Scuseria GE, Ernzerhof M (2006). Hybrid functionals based on a screened Coulomb potential (vol 118, pg 8207, 2003). J Chem Phys.

[29] Yanai T, Tew DP, Handy NC (2004). A new hybrid exchange-correlation functional using the Coulomb-attenuating method (CAM-B3LYP). Chem Phys Lett.

[30] Peverati R, Truhlar DG (2011). Improving the accuracy of hybrid meta-GGA density functionals by range separation. J Phys Chem Lett.

[31] Chai JD, Head-Gordon M (2008). Systematic optimization of long-range corrected hybrid density functionals. J Chem Phys.

[32] Iikura H, Tsuneda T, Yanai T, Hirao K (2001). A long-range correction scheme for generalized-gradient-approximation exchange functionals. J Chem Phys.

[33] Song JW, Watson MA, Hirao K (2009). An improved long-range corrected hybrid functional with vanishing Hartree–Fock exchange at zero interelectronic distance, LC2gau-BOP. J Chem Phys.

[34] Vydrov OA, Scuseria GE (2006). Assessment of a long-range corrected hybrid functional. J Chem Phys.

[35] Jaramillo J, Scuseria GE, Ernzerhof M (2003). Local hybrid functionals. J Chem Phys.

[36] Arbuznikov AV, Kaupp M (2008). What can we learn from the adiabatic connection formalism about local hybrid functionals?. J Chem Phys.

[37] Bahmann H, Rodenberg A, Arbuznikov AV, Kaupp M (2007). A thermochemically competitive local hybrid functional without gradient corrections. J Chem Phys.

[38] Haunschild R, Janesko BG, Scuseria GE (2009). Local hybrids as a perturbation to global hybrid functionals. J Chem Phys.

[39] Janesko BG, Scuseria GE (2007). Local hybrid functionals based on density matrix products. J Chem Phys.

[40] Janesko BG, Scuseria GE (2008). Parameterized local hybrid functionals from density-matrix similarity metrics. J Chem Phys.

[41] Johnson ER (2014). Local-hybrid functional based on the correlation length. J Chem Phys.

[42] Haunschild R, Scuseria GE (2010). Range-separated local hybrids. J Chem Phys.

[43] Henderson TM, Janesko BG, Scuseria GE, Savin A (2009). Locally range-separated hybrids as linear combinations of range-separated local hybrids. Int J Quantum Chem.

[44] Arbuznikov AV, Kaupp M (2012). Importance of the correlation contribution for local hybrid functionals: range separation and self-interaction corrections. J Chem Phys.

[45] Grimme S (2006). Semiempirical hybrid density functional with perturbative second-order correlation. J Chem Phys.

[46] Hedegard ED, Heiden F, Knecht S, Fromager E, Jensen HJA (2013). Assessment of charge-transfer excitations with time-dependent, range-separated density functional theory based on long-range MP2 and multiconfigurational self-consistent field wave functions. J Chem Phys.

[47] Janesko BG, Henderson TM, Scuseria GE (2009). Long-range-corrected hybrids including random phase approximation correlation. J Chem Phys.

[48] Furche F (2008). Developing the random phase approximation into a practical post-Kohn-Sham correlation model. J Chem Phys.

[49] Furche F, Van Voorhis T (2005). Fluctuation-dissipation theorem density-functional theory. J Chem Phys.

[50] Eshuis H, Furche F (2011). A parameter-free density functional that works for noncovalent interactions. J Phys Chem Lett.

[51] Goll E, Werner HJ, Stoll H (2005). A short-range gradient-corrected density functional in long-range coupled-cluster calculations for rare gas dimers. Phys Chem Chem Phys.

[52] Goll E, Werner HJ, Stoll H, Leininger T, Gori-Giorgi P, Savin A (2006). A short-range gradient-corrected spin density functional in combination with long-range coupled-cluster methods: application to alkali-metal rare-gas dimers. Chem Phys.

[53] Garza AJ, Bulik IW, Henderson TM, Scuseria GE (2015). Range separated hybrids of pair coupled cluster doubles and density functionals. Phys Chem Chem Phys.

[54] Goerigk L, Grimme S (2014). Double-hybrid density functionals. Wiley Interdiscip Rev Comput Mol Sci.

[55] Chai JD, Head-Gordon M (2009). Long-range corrected double-hybrid density functionals. J Chem Phys.

[56] Burke K (2012). Perspective on density functional theory. J Chem Phys.

[57] Pribram-Jones A, Gross DA, Burke K (2015) DFT: a theory full of holes? In: Johnson MA, Martinez TJ (eds) Ann Rev Phys Chem 66:283–304. doi:10.1146/annurev-physchem-040214-121420 10.1146/annurev-physchem-040214-121420 25830374

[58] Abbott A, Cyranoski D, Jones N, Maher B, Schiermeier Q, Van Noorden R (2010). Do metrics matter?. Nature.

[59] Van Noorden R (2010). A profusion of measures. Nature.

[60] Barth A, Marx W (2012). Stimulation of Ideas through compound-based bibliometrics: counting and mapping chemical compounds for analyzing research topics in chemistry, physics, and materials science. Chemistryopen.

[61] Marx W, Bornmann L, Barth A, Leydesdorff L (2014). Detecting the historical roots of research fields by reference publication year spectroscopy (RPYS). J Assoc Inf Sci Technol.

[62] Goedecke C, Hillebrecht P, Uhlemann T, Haunschild R, Frenking G (2009). The Dewar–Chatt–Duncanson model reversed - Bonding analysis of group-10 complexes (PMe3)(2)M-EX3 (M = Ni, Pd, Pt; E = B, Al, Ga, In, Tl; X = H, F, Cl, Br, I). Can J Chem Rev Can Chim.

[63] Thor A, Marx W, Leydesdorff L, Bornmann L (2016) Introducing CitedReferencesExplorer (CRExplorer): a program for reference publication year spectroscopy with cited references disambiguation. http://arxiv.org/abs/1601.01199. Accessed 1 Oct 2016

[64] Haunschild R, Bornmann L, Marx W (2016). Climate change research in view of bibliometrics. PLoS ONE.

[65] Noel Y, De La Pierre M, Zicovich-Wilson CM, Orlando R, Dovesi R (2014). Structural, electronic and energetic properties of giant icosahedral fullerenes up to C6000: insights from an ab initio hybrid DFT study. Phys Chem Chem Phys.

[66] Holec D, Hartmann MA, Fischer FD, Rammerstorfer FG, Mayrhofer PH, Paris O (2010). Curvature-induced excess surface energy of fullerenes: density functional theory and Monte Carlo simulations. Phys Rev B.

